# Fulgence Raymond: from rural life and veterinary medicine to Charcot's successor at La Salpêtrière Hospital

**DOI:** 10.1055/s-0044-1789203

**Published:** 2024-08-26

**Authors:** Hélio A. G. Teive, Catarina Dantas Correa, Léo Coutinho, Carlos Henrique Ferreira Camargo, Olivier Walusinski

**Affiliations:** 1 Universidade Federal do Paraná, Departamento de Clínica Médica, Serviço de Neurologia, Curitiba PR, Brazil.; 2 Universidade Federal do Paraná, Programa de Pós-Graduação em Medicina Interna, Disciplina de Doenças Neurodegenerativas, Curitiba PR, Brazil.; 3 Clínica Privada, Brou, France.

**Keywords:** History of Medicine, Neurology, Veterinary Medicine, Salpêtrière Hospital, História da Medicina, Neurologia, Medicina Veterinária, Hospital de La Salpêtrière

## Abstract

This paper provides a historical overview of Professor Fulgence Raymond, Charcot's eldest pupil, who was chosen as his successor. It explores Raymond's origins as a veterinary surgeon, his evolution as a neurologist under Charcot's mentorship, and his tenure as the professor's successor at the La Salpêtrière Hospital in Paris, France, from 1894 to 1910.

## Resumo

O presente artigo oferece um perfil histórico do professor Fulgence Raymond, que foi o pupilo mais velho do Professor Jean-Martin Charcot, é apresentado, destacando-se a origem de Raymond como cirurgião veterinário, sua carreira como médico neurologista sob supervisão de Charcot e, finalmente, a sua atuação como sucessor do professor , na cadeira de doenças do sistema nervoso do Hospital de La Salpêtrière, em Paris, França, entre os anos de 1894 e 1910.

## INTRODUCTION

During the tenure of Jean-Martin Charcot (1825–1893) as the head of the Neurology Service at La Salpêtrière Hospital in Paris, France, from 1862 until his passing in 1893, he mentored 32 *internes* (house officers), many of whom attained international acclaim. 1 2 Among these, 5 were appointed as Charcot's *chefs de clinique* (specialist registrars), starting in 1882 ( Table 1 ). 1 Fulgence Raymond (1844–1910) was 31 years old when he served as a house officer at La Salpêtrière in 1875, making him professor Charcot's eldest pupil. 1 2 3

**Table 1:** Charcot's *internes* (house officers) and *chefs de clinique* (specialist registrars)

Year	Interne	Chef de clinique
1862	1. Henri Soulier 1834–1921	
1863	2. Victor Cornil 1837–1908	
1864	3. Charles Bouchard 1837–1915	
1865	4. Jules Cotard 1840–1889	
1866	Charles Bouchard 1837–1915	
1867	5. Raphael Lépine 1840–1919	
1868	6. Désiré Magloire Bourneville 1840–1909	
1869	7. Alix Joffroy 1844–1908	
1870	8. Jules-Aimé Michaud	
1871	Jules-Aimé Michaud	
1872	9. Albert Gombault 1844–1904	
1873	10. Georges Debove 1845–1920	
1874	11. Antoine-Auguste Pierret 1845–1920	
1875	12. Fulgence Raymond 1844–1910	
1876	13. Albert Pitres 1848–1928	
1877	14. Paul Oulmont 1849–1917	
1878	15. Paul Richer 1849–1933	
1879	16. Edouard Brissaud 1852–1909	
1880	17. Gilbert Ballet 1853–1916	
1881	18. Charles Féré 1852–1907	
1882	19. Pierre Marie 1853–1940	1. Gilbert Ballet 1853–1916
1883	20. Antoine Bernard 1853–1891	2. Pierre Marie 1853–1940
1884	21. Gilles de la Tourette 1857–1904	
1885	22. Georges Guinon 1859–1932	3. Joseph Babinski 1857-1932
1886	23. Paul Berbez 1859-?	
1887	24. Paul Blocq 1860–1896	4. Gilles de la Tourette 1857–1904
1888	25. Ernest Huet 1858–1917	
1889	26. Adolphe Dutil 1862–1899(?)	5. Georges Guinon 1859–1932
1890	27. Achille Souques 1860–1944	
1890	28. Emile Parmentier 1860–1940	
1891	29. Jean-Baptiste Charcot 1867–1936	6. Adolphe Dutil 1862–1899(?)
	30. Louis Hallion 1862–1940	
1892	31. Jean-Félix Guyon 1864–1907	
	32. Henri Lamy 1864–1909	

The objective of this historical account is to chronicle Raymond's trajectory from his upbringing in the countryside to his ascension as a neurologist, ultimately assuming the role of head of the Neurology Service at La Salpêtrière, succeeding Charcot.

### Fulgence Raymond – a brief biography

**Figure 1:**
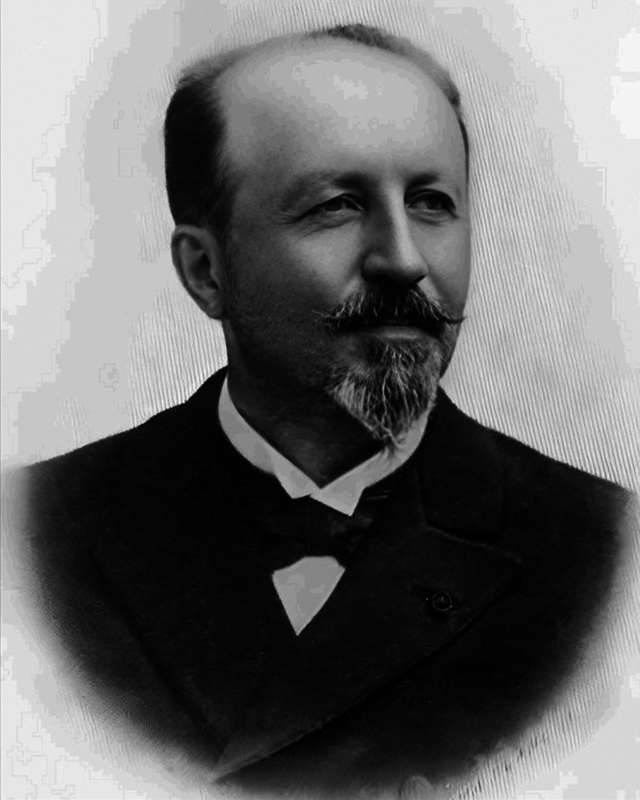
Professor Fulgence Raymond (1844–1910). Source: Wikimedia Commons, April 2024.

Fulgence Raymond ( Figure 1 ) was born on September 29, 1844, in the small town of Saint-Christophe-sur-le-Nais, Indre-et-Loire, in central France. He was the son of Créon Raymond (1814–1884) and Justine Police (1820–1882), both farmers from humble origins. 1 3 4 Influenced by his father, Raymond enrolled in the École Impériale Vétérinaire de Maisons-Alfort in 1861. Graduating in 1865, he quickly rose to become professor and head of the anatomy and physiology department by 1866. 1 3 4 5 6 Subsequently, he showcased his expertise as a veterinarian at the École de Cavalerie, in Saumur, demonstrating remarkable skill in both veterinary medicine and equestrianism. 1 3 4

Despite finding satisfaction in his veterinary career, Raymond recognized the limited recognition and low remuneration it offered. Thus, he resolved to pursue medical studies. Raymond's transition to medicine proved successful, and in 1868, he commenced his medical education at the École de Médecine in Paris, ultimately earning his medical degree in 1877. 1 2 3 4 5 6 During the Franco-Prussian War, from 1870 to 1871, Raymond served in the ambulance service in Paris. Tragically, following the war, Raymond experienced profound loss when his wife, Louise Rochut (1842–1872), succumbed to tuberculosis. He remarried in 1887 to Marie-Louise Lodoiska Moreau (1850–1945). 1 4

Raymond was honored with the gold medal of the 1875 internship and defended his thesis on hemichorea, hemianesthesia, and tremors in 1876 under the supervision of Charcot. 1 2 3 4 5 6 Subsequently, he served as a *chef de clinique* under Germain Sée (1818–1896) in 1877, obtained the position of *médecin des hôpitaux* (hospital doctor) in the following year, and achieved habilitation in 1880. Raymond conducted rotations in various hospitals across Paris, including neurology services under Alfred Vulpian (1826–1887) and Charcot at La Salpêtrière, where he was appointed as an assistant professor at the École de Médecine in 1880. 1 3 4 5 6

Colleagues and biographers characterize Raymond as affable, serene, optimistic, and content. He harbored a keen interest in archaeology and enjoyed hunting, unlike his mentor. 1 2 3 4 5 6 Embarking on a prolific scientific journey, Raymond authored numerous papers, establishing himself as one of Charcot's most accomplished disciples. He passed away on September 28, 1910, in Paris. 1 2 3 4 5 6 7 8

### Fulgence Raymond's scientific output

Under the profound influence of the La Salpêtrière school, Raymond made significant contributions across various domains of neurology. His publications encompassed a wide array of topics, including case reports and studies on neuroanatomy, neuropathology, aphasia, myelopathies, locomotor ataxia, familial essential tremor, neuropsychology (in collaboration with Pierre Janet), and acute polyradiculoneuropathies (Guillain-Barré-Strohl syndrome). 1 3 4 5 6 7 8 Notably, Raymond played a pivotal role in elucidating the Raymond-Cestan syndrome, a crossed-segmental condition of the brainstem resulting from lesions of the vertebrobasilar system. It is characterized by internuclear ophthalmoplegia, hemicerebellar syndrome, and contralateral hemiparesis, also known as *syndrome protubérantiel supérieur* . 1 3 4 5 6 8 9 10

**Figure 2:**
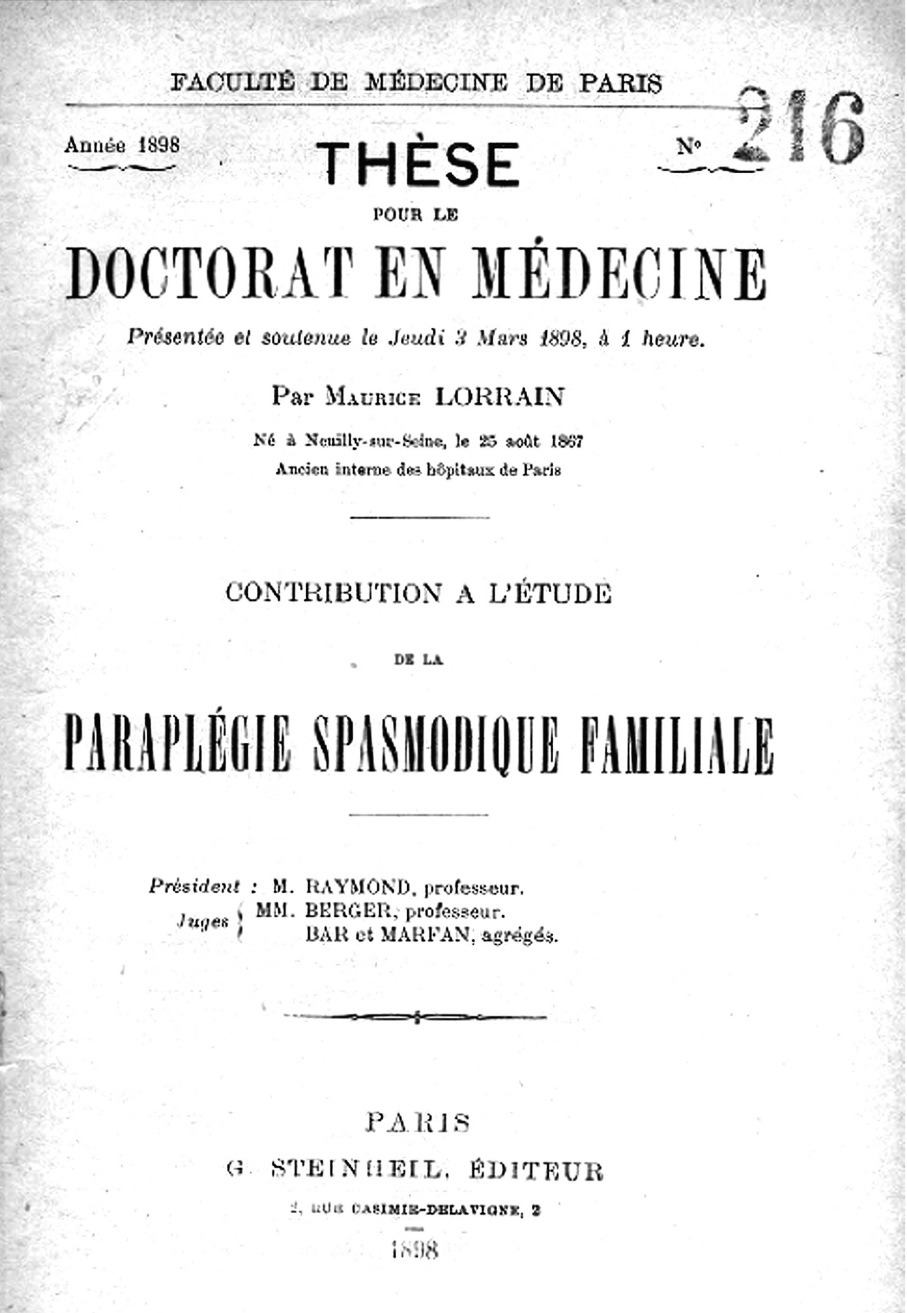
Maurice Lorrain's thesis on *Paraplégie Spasmodique Familiale* under the direction of Professor Fulgence Raymond (1898). Source: courtesy of Dr. Olivier Walusinski.

Furthermore, his studies on hereditary spastic paralysis (HSP), conducted in 1895, significantly advanced the understanding of the disease. Maurice Lorrain (1867–1956), an intern in Raymond's service, published his thesis in 1898 entitled “L'étude de la paraplégie spasmodique familiale” under Raymond's supervision ( Figure 2 ). 1 3 4 5 6 8 11 12 13 Throughout his scientific career, Raymond maintained a keen interest in investigating familial spastic paraplegia, yet, curiously, his name was not attributed to the disease, which was instead defined as Strümpell-Lorrain HSP. 1 3 11 12 13

Raymond authored several books, including six editions of the renowned work entitled “ *Leçons sur les maladies du système nerveux.”* 14 Additionally, he held esteemed editorial positions in leading scientific journals of his era in France. He served as editor of the Epilepsia journal at the University of Oxford in the United Kingdom, where he was conferred an honorary Doctor of Science (D.Sc.) title. Following his international acclaim, Raymond was elected to the Académie Nationale de Médecine and became one of the founding members of the Société de Neurologie de Paris. 1 3 4 5 7 8 15

### Raymond and the Charcot's succession

Following Charcot's unexpected demise in 1893, Édouard Brissaud (1852–1909) assumed an interim position as the head of the *Chaire de Clinique des Maladies du Système Nerveux* at La Salpêtrière for 1 year. Subsequently, after an open competition in 1894, Fulgence Raymond was selected as the head. 1 2 3 4 5 6 The pool of candidates included Brissaud, regarded as one of Charcot's most exceptional pupils but deemed relatively young for the role, and Jules Dejerine (1849–1917), a clinical neurologist and distinguished researcher with significant scientific acclaim, albeit known as a staunch adversary of Charcot and his school. Gilles de la Tourette and Babinski, having not passed the *agrégation* examination, were ineligible for consideration for the vacant position. Pierre Marie, who would eventually assume the role in 1917, held the position of *professeur agrégé* (Associate Professor) since 1889 but considered himself too young for the contention. 1 2 3 4 5 6 Raymond's seniority was pivotal in his selection as Charcot's successor, alongside his esteemed neurological expertise, capabilities, and extensive scholarly contributions. 1 2 3 4 5 6 8

Fulgence Raymond, initially a country veterinarian, transitioned to a distinguished academic trajectory in human medicine. His prolific scholarly contributions in neurology, heavily influenced by the legacy of Charcot's school, underscore his remarkable journey. Political considerations also played a role in his selection as Charcot's successor at the École de Médecine in Paris, France, a position he held from 1894 to 1910.
